# Dysregulated Circulating Dendritic Cell Function in Ulcerative Colitis Is Partially Restored by Probiotic Strain *Lactobacillus casei* Shirota

**DOI:** 10.1155/2013/573576

**Published:** 2013-07-18

**Authors:** Elizabeth R. Mann, Jialu You, Verena Horneffer-van der Sluis, David Bernardo, Hafid Omar Al-Hassi, Jon Landy, Simon T. Peake, Linda V. Thomas, Cheng T. Tee, Gui Han Lee, Ailsa L. Hart, Parveen Yaqoob, Stella C. Knight

**Affiliations:** ^1^Antigen Presentation Research Group, Imperial College London, Northwick Park and St. Mark's Campus, Level 7W St. Mark's Hospital, Watford Road, Harrow HA1 3UJ, UK; ^2^Department of Food and Nutritional Sciences, University of Reading, Reading, UK; ^3^Northwick Park Institute for Medical Research, Harrow, UK; ^4^St. Mark's Hospital, North West London Hospitals NHS Trust, Harrow, UK; ^5^Yakult UK Ltd., West End Road, South Ruislip, UK

## Abstract

*Background*. Dendritic cells regulate immune responses to microbial products and play a key role in ulcerative colitis (UC) pathology. We determined the immunomodulatory effects of probiotic strain *Lactobacillus casei* Shirota (LcS) on human DC from healthy controls and active UC patients. *Methods*. Human blood DC from healthy controls (control-DC) and UC patients (UC-DC) were conditioned with heat-killed LcS and used to stimulate allogeneic T cells in a 5-day mixed leucocyte reaction. *Results*. UC-DC displayed a reduced stimulatory capacity for T cells (*P* < 0.05) and enhanced expression of skin-homing markers CLA and CCR4 on stimulated T cells (*P* < 0.05) that were negative for gut-homing marker *β*7. LcS treatment restored the stimulatory capacity of UC-DC, reflecting that of control-DC. LcS treatment conditioned control-DC to induce CLA on T cells in conjunction with *β*7, generating a multihoming profile, but had no effects on UC-DC. Finally, LcS treatment enhanced DC ability to induce TGF*β* production by T cells in controls but not UC patients. *Conclusions*. We demonstrate a systemic, dysregulated DC function in UC that may account for the propensity of UC patients to develop cutaneous manifestations. LcS has multifunctional immunoregulatory activities depending on the inflammatory state; therapeutic effects reported in UC may be due to promotion of homeostasis.

## 1. Introduction

Interactions between the host and microbiota play a crucial role in mucosal immune homeostasis [1]. Certain strains of lactic-acid producing bacteria are classed as probiotics because their consumption is associated with health benefits, which are mediated via the gut. The current probiotic definition is “live microorganisms which when administered in adequate amounts confer a healthy benefit on the host” [2]. Probiotic bacteria are most frequently of the *Lactobacillus *or *Bifidobacterium *species and usually species that can be found in the normal commensal microbiota. Probiotics can be effective in treating some patients with inflammatory bowel disease (IBD) [3–7], but the details of which strains confer benefit and their mechanisms of action are only slowly being defined.

Ulcerative colitis (UC) and Crohn's disease (CD), collectively termed inflammatory bowel disease (IBD), result from a dysregulated response of the mucosal immune system to components of the luminal microbiota and breakdown of immune tolerance in individuals who are genetically predisposed to the disease. These processes lead to “inappropriate” activation of mucosal T cells and production of inflammatory mediators [8–11].

Dendritic cells (DC) recognize and respond to bacteria and bacterial products and generate primary T cell responses. DC also determine whether T cell responses generated are immunogenic or tolerogenic [12–14]. In particular, intestinal DC maintain the delicate balance in the gut between immunogenicity against invading pathogens and tolerance of the commensal microbiota [15]; alterations in intestinal DC have been found in IBD [15, 16]. The effects of probiotic bacteria on DC, which are so pivotal in early bacterial recognition, tolerance induction, and shaping T cell responses, are likely to be central in immunomodulation by these bacteria and are likely to partially account for the reported efficacy of probiotics in IBD [3–7].

IBD is associated with a variety of extraintestinal manifestations (EIM), with up to a third of IBD patients developing cutaneous manifestations [17]. The causes of EIM are poorly understood, but it has been suggested that compartmentalisation of inflammatory processes to different organs (e.g., the intestines, skin, or liver) may be linked to homing and trafficking of immune cells [18]. Indeed, dysregulated lymphocyte trafficking has been reported in both UC and CD [19–22].

Homing properties are imprinted on T cells upon stimulation by DC, to localise immune responses to specific tissues [23–26]. Effector T cells migrating to intestinal sites express high levels of gut-homing molecule *α*_4_ *β*_7_ [27], with its ligand MAdCAM-1 being constitutively expressed by postcapillary endothelial cells in the small intestine [28] and colonic lamina propria [29]. Skin T cells express E- and P-selectin ligands including cutaneous lymphocyte-associated antigen (CLA) [30] and CCR4 [31]. The occurrence of EIM associated with IBD indicates a systemic disease, rather than immune dysregulation confined to intestinal sites; however it is currently unclear whether alterations in *circulating *DC occur in IBD patients, including DC ability to imprint specific homing properties on stimulated T cells. Trafficking of immune cells is an area yet to be investigated regarding specific mechanisms of action of immunomodulation by probiotics or dysregulated DC function in IBD.

The strain-specific nature of the immunomodulatory effects of probiotics is well established; some *Lactobacillus *strains induce production of regulatory cytokines, suppress Th1 responses, and are thought to be involved in oral tolerance. In contrast, other strains induce production of pro inflammatory cytokines. However, human intervention studies have shown a variety of beneficial immunomodulatory effects associated with consumption of the probiotic bacterial strain *Lactobacillus casei *Shirota (LcS) specifically, including significant improvement in UC disease activity index (UCDAI) scores in patients with mild-moderate UC administered LcS orally for 8 weeks, compared to pretreatment and also patients on conventional therapy. The same study demonstrated that LcS reduces production of IL-6 from peripheral blood mononuclear cells (PBMC) *in vitro *[32]. Other studies demonstrate reduction of gingival inflammation [33] and downregulation of allergic responses [34] following consumption of LcS. To this end, we aimed to determine whether systemic changes exist between healthy controls and patients with active UC, regarding the ability of circulating (blood-enriched) DC to generate effector T cell responses and imprint specific homing properties on T cells stimulated. We also aimed to study the immunomodulatory effects of probiotic strain LcS on such DC.

## 2. Materials and Methods

### 2.1. Human Peripheral Blood

Human peripheral blood was collected from healthy volunteers with no known autoimmune or inflammatory diseases, allergies or malignancies (*n* = 8) or from patients with active UC following informed consent (*n* = 6). Disease activity for UC was assessed using the UC disease activity index (UCDAI); patients scoring UCDAI 4–12, alongside diagnosis from clinical parameters, radiographic studies, and endoscopic and histological criteria, were defined as active UC. Patients were treatment naïve or on minimal treatment: 5-aminosalicylic acid (5ASA) and/or azathioprine (AZA). Peripheral blood mononuclear cells (PBMC) were obtained by centrifugation over Ficoll-Paque Plus (Amersham Biosciences, Chalfont St. Giles, UK). Human blood-enriched DC (low density cells or LDC) were obtained following NycoPrep centrifugation of overnight cultured PBMC. These cells were 98%–100% HLA-DR^+^, with morphological characteristics of DC (both at optical microscopy and electron microscopy), and are potent stimulators of naïve T cells. Blood LDC have been characterised in detail in previous studies from our laboratory [35, 36] and will be referred to as blood DC in this study.

### 2.2. Conditioning of Human Blood DC by LcS

Stock culture of LcS (Yakult Honsha Co. Ltd., Tokyo, Japan) was cultured at 37°C for 24 hours in MRS broth and grown on MRS agar (Oxoid, Hampshire, UK) for 48 hours at 37°C in an anaerobic cabinet (MACS MG 1000; Don Whitley Scientific, West Yorkshire, UK) with a gas mixture of 10% H_2_, 10% CO_2_, and 80% N_2_ by volume. For liquid culture, one pure colony was taken from an MRS nutrient agar plate and grown overnight in 10 mL of prereduced MRS broth (Oxoid) with 0.05% L-cysteine hydrochloride (Sigma, Dorset, UK) in a shaking incubator at 37°C; 0.5 mL of the overnight culture was inoculated into another 10 mL MRS broth. The bacteria were harvested in the exponential phase, resuspended in phosphate-buffered saline (PBS; Oxoid), centrifuged twice at 1960 g (Sanyo/MSE Micro Centaur, Haverhill, USA) for 5 minutes, and resuspended at the required concentration in RPMI 1640 containing 0.75 mM L-glutamine. Bacteria were then heat-killed with viability checks done to make sure that no bacteria survived, and varying concentrations (1 × 10^5^, 1 × 10^6^, or 1 × 10^7^) of heat-killed LcS were used to condition 2.5 × 10^5^ blood DC in 1 mL total volume of complete medium (Dutch modification RPMI 1640 containing 2 mM glutamine, 10% fetal calf serum, and 100 U/mL penicillin/streptomycin) for 24 hours. Control conditions involved conditioning DC with complete medium only for 24 hours. Following conditioning, DC were washed and used in a mixed-leucocyte reaction (MLR) with allogeneic T cells.

### 2.3. Enrichment of Blood T Cells

PBMC were suspended in MiniMACS buffer (PBS containing 0.5% BSA and 2 mM EDTA) and T cells were enriched by depletion of CD14^+^, CD19^+^, and HLA-DR^+^ cells with immunomagnetic beads (Miltenyi Biotech, Bisley, UK) following manufacturer's instructions.

### 2.4. T cell Proliferation Assay

Carboxyfluorescein diacetate succinimidyl ester (CFSE, Invitrogen Ltd, UK) labelled T cells (4 × 10^5^/well) were incubated for 5 days in U-bottomed 96-well microtitre plates with enriched, previously conditioned, allogeneic DC at 0%, 1%, 2%, or 3% in a mixed leukocyte reaction (MLR). Cells were recovered and CFSE^lo^ proliferating cells identified and quantified by flow cytometry.

### 2.5. Antibody Labelling

Monoclonal antibodies with the following specificities and conjugations were used: CLA-FITC (HECA-452), *β*7 integrin-PE (FIB504), IL-12 (p40/p70)-PE (C11.5), IL-17A-PE (SCPL1362), CD3-PerCPCy5.5 (SK7), CD3-PeCy5 (UCHT1), IL-10-APC (JES3-19F1), IFN*γ* (25723.11), CLA-biotin (HECA-452), and Streptavidin-APC were purchased from BD Biosciences (Oxford, UK); CCR9 (either FITC or APC) (112509), CCR7-PE (150503), CCR10-APC (314315), CCR4-APC (205410), and TGF*β* (IC388P) were purchased from R&D Systems (Abingdon, UK). Appropriate isotype-matched control antibodies were purchased from the same manufacturers. After the staining, cells were fixed with 1% paraformaldehyde in 0.85% saline and stored at 4°C prior to acquisition on the flow cytometer, within 48 hours.

### 2.6. Flow Cytometry and Data Analysis

Data were acquired on a FACSCanto II cytometer (BD Biosciences) and analysed using WinList 5.0 software (Verity, ME, US). Proportions of positive cells were measured by subtracting the appropriate isotype-matched control staining from test histogram using superenhanced D_max⁡_ (SED) normalised subtraction.

### 2.7. Cytokine Analysis

The intracellular cytokine production by stimulated T cells after MLR was measured using superenhanced D_max⁡_ (SED) normalised subtraction upon data analysis following incubation +/− monensin, T cell permeabilisation, antibody labeling, and flow cytometry.

### 2.8. Statistical Analyses

Data are presented as mean and standard errors. Two-way repeated measures ANOVA and two-tailed paired *t*-tests were applied as stated in the figure legends. In the case of multiple comparisons, subsequent *ad hoc *Bonferroni correction was applied. *P* < 0.05 was considered significant.

## 3. Results

### 3.1. Characteristics of Human DC Function in UC 

#### 3.1.1. Reduced T cell Stimulatory Capacity of DC in UC

**Figure 1:**
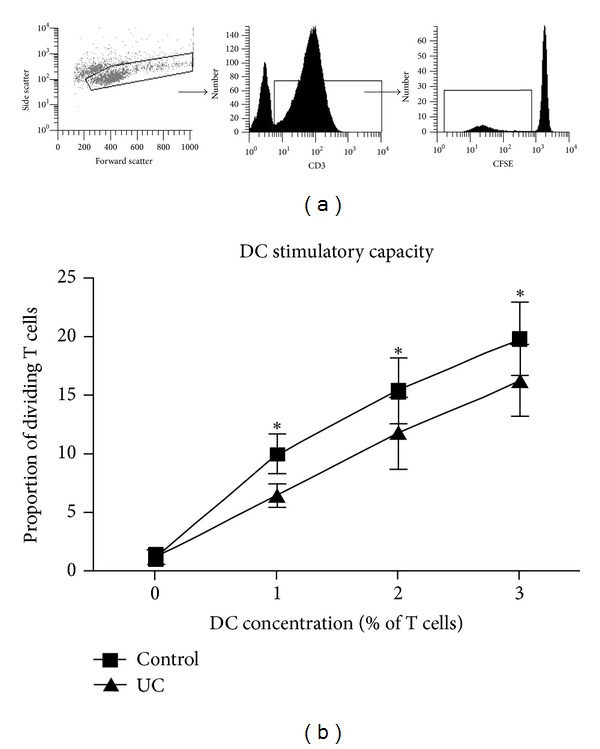
Restricted DC stimulatory capacity in UC. (a) Identification of dividing T cells following mixed leucocyte reaction (MLR) according to flow cytometry forward and side scatter dot plot and subsequent CD3 and CFSE histograms, respectively. (b) Dose response T cell proliferation following MLR. Results are displayed as mean ± SEM (*n* = 6). Base-level proliferation is shown as proportion of dividing T cells with no DC (0%). After paired two-way ANOVA analysis (corrected with Bonferroni correction for multiple comparisons), the DC concentration was statistically significant in both cases (*P* < 0.01), that is, a dose response occurred in both cases. UC-DC were less stimulatory than control DC (*P* < 0.05 at 1%, 2%, and 3% DC).

We analysed DC stimulation of T cells in a 5-day mixed leucocyte reaction (MLR). T cells from the same donor (a separate, healthy control) were stimulated by DC from healthy controls and UC patients, within the same experiments. DC stimulated a strong, dose-dependent proliferative response in both healthy controls and UC patients; dividing T cells were identified as CFSE^lo^ CD3^+^ lymphocytes, by flow cytometry (Figure 1(a)). However, DC from UC patients (UC-DC) stimulated a significantly weaker proliferation of the same CFSE-labelled T cells compared with DC from healthy controls (control DC; Figure 1(b)).

#### 3.1.2. DC in UC Exhibit an Enhanced Ability to Imprint Skin-Homing Properties on Effector T cells

**Figure 2:**
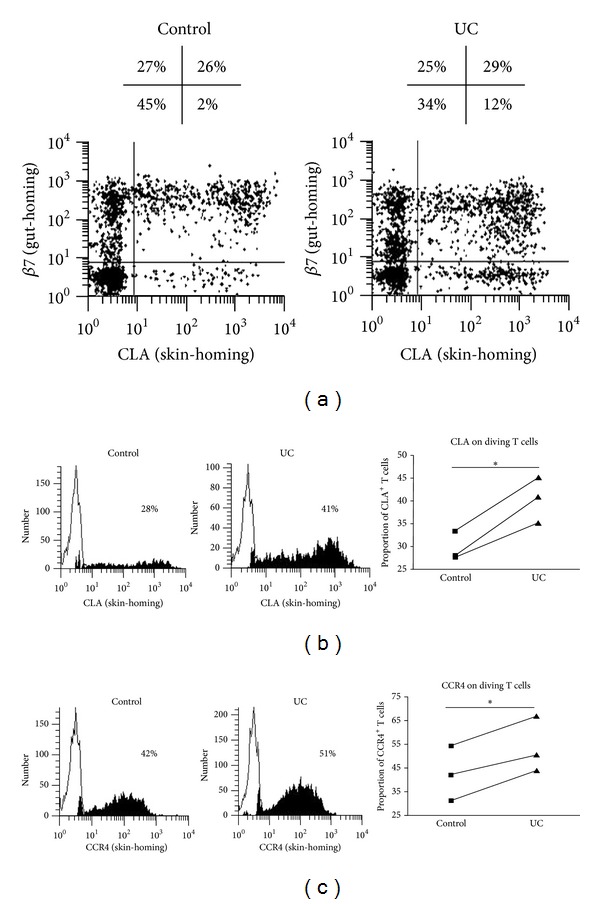
UC-DC enhanced expression of skin-homing molecules on stimulated T cells. (a) CLA/*β*7 dot plots of dividing T cells, following stimulation by 3% control or UC-DC. Numbers over each dot plot represent proportion of dividing T cells expressing *β*7 only, *β*7 and CLA, CLA only, or either *β*7 or CLA. (b) Histograms of CLA expression by dividing T cells, following stimulation by 3% control or UC-DC. On the right, summary graph of all experiments (*n* = 3). (c) Histograms of CCR4 expression by dividing T cells, following stimulation by 3% control or UC-DC. On the right, summary graph of all experiments (*n* = 3). Paired *t*-test was applied; *P* value <0.05 was considered statistically significant (*P < 0.05). All representative histograms/dot plots are from a single experiment representative of 3 independent experiments performed with similar results. Filled histograms represent positive staining; empty histograms represent background staining.

We have previously demonstrated that T cells within fresh PBMC expressed *either *gut-homing molecule *β*7 or skin-homing molecule CLA; the majority expressed *β*7 only. Freshly purified T cells exhibited the same homing profile, prior to coculture with allogeneic DC [37]. After culture, the expression of *β*7 on dividing T cells (CFSE^lo^) was the default pathway; T cells stimulated by both control and UC-DC maintained *β*7 expression, as did unstimulated T cells. In contrast, CLA expression was induced on dividing T cells by both control and UC-DC so that substantial numbers of T cells were identified as double positive for CLA and *β*7 following stimulation (due to inherent high expression of *β*7 in all conditions; Figure 2(a)). However, UC-DC exhibited an enhanced ability to prime skin-homing T cells, significantly increasing the proportion of total CLA^+^ T cells (Figure 2(b)) and the proportion of T cells expressing skin-homing molecule CCR4 (Figure 2(c)) within the stimulated population.

### 3.2. Effects of LcS Treatment on Dendritic Cell Function

#### 3.2.1. LcS Restored T cell Stimulatory Capacity of Dendritic Cells in UC

**Figure 3:**
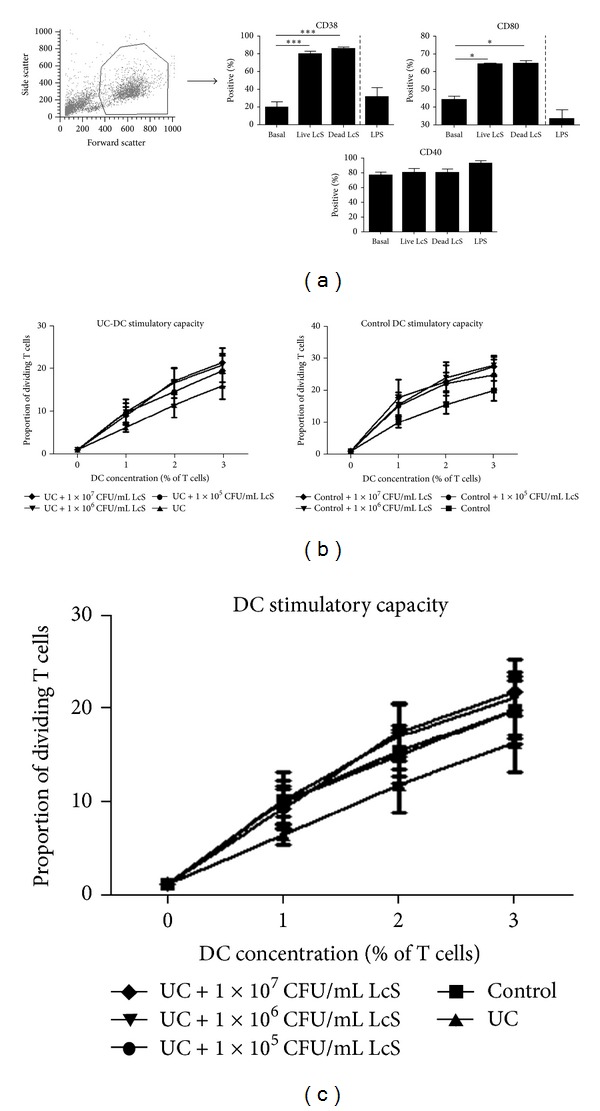
LcS restored “normal” DC stimulatory capacity in UC. (a) Identification of blood-enriched DC according to flow cytometry forward and side scatter plot and summary graphs representing mean ± SEM proportions of DC expressing CD40 and CD86 following conditioning with medium only, live LcS or dead LcS (*n* = 3). Separate experiments were carried out conditioning DC with LPS (*n* = 3). (b) Dose response T cell proliferation following MLR with control- and UC-DC (*n* = 6). After paired two-way ANOVA analysis (corrected with Bonferroni correction for multiple comparisons), the DC concentration was statistically significant in all cases (*P* < 0.01). Control- and UC-DC stimulatory capacity was increased following LcS conditioning at LcS concentrations of 1 × 10^5^ (control: *P* < 0.05 at 2%, UC: *P* < 0.01 at 1%, *P* < 0.05 at 2%, *P* < 0.01 at 3% DC), 1 × 10^6^ (control: *P* < 0.01 at 2% and 3%, UC: *P* < 0.05 at 2%, *P* < 0.01 at 3% DC), and 1 × 10^7^ (control: *P* < 0.01 at 1%, 2%, and 3%, UC: *P* < 0.01 at 2% and 3%) CFU/mL. (c) There were no significant differences between the stimulatory capacity of control and LcS-conditioned UC-DC at any LcS concentration.

Optimisation experiments on healthy control DC determined no significant differences between live or heat-killed (HK) LcS regarding ability to enhance DC activation/maturation markers CD80 and CD83; both live and HK LcS significantly enhanced CD80 and CD83 expression (Figure 3(a)). Therefore HK LcS was used for all further experiments.

We analysed DC stimulation of T cells in a 5-day mixed leucocyte reaction (MLR) following DC conditioning with complete medium only or varying concentrations of HK LcS (1 × 10^5^, 1 × 10^6^, or 1 × 10^7^ CFU/mL). A significant, dose-dependent increase in DC stimulatory capacity was observed upon LcS conditioning of both control- and UC-DC (Figure 3(b)). Following LcS conditioning, UC-DC levels of stimulation were restored to “normal” levels, similar to that of control DC (Figure 3(c)).

#### 3.2.2. LcS Conditioned DC to Imprint Skin-Homing Properties on T Cells in Healthy Controls but Not UC Patients

**Figure 4:**
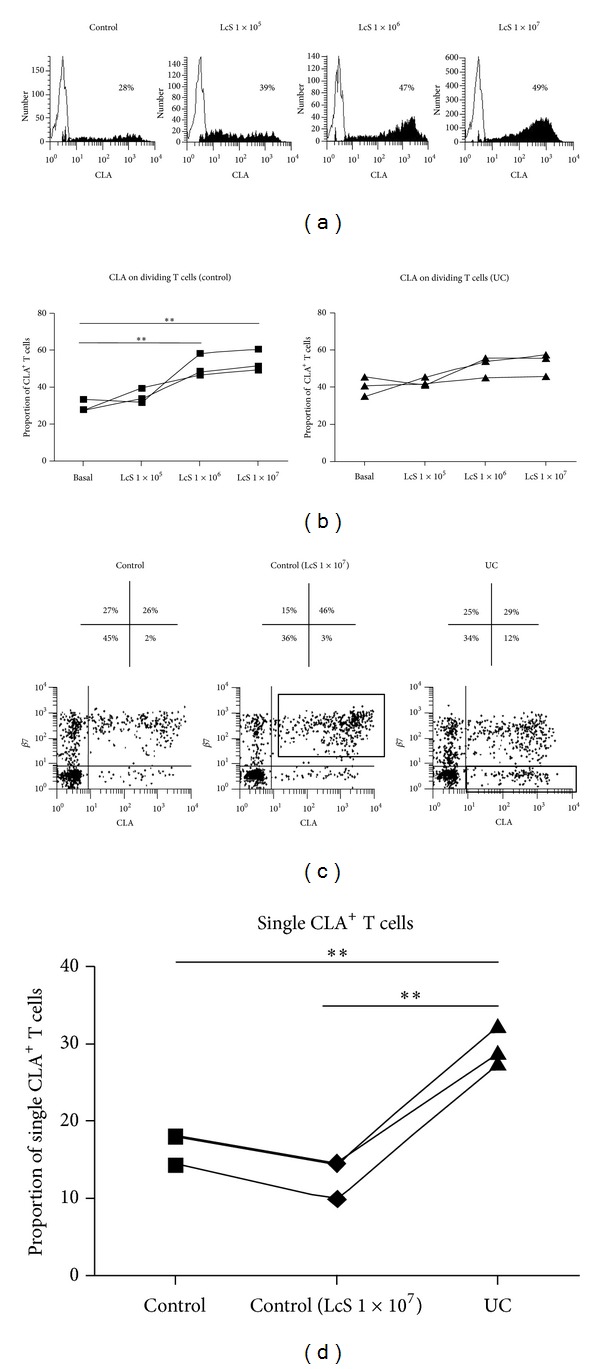
LcS conditioning of DC enhanced expression of CLA on stimulated T cells in healthy controls but not UC. (a) Histograms of CLA expression by dividing T cells, following stimulation by 3% DC from healthy control (no LcS) and after conditioning with 1 × 10^5^/1 × 10^6^/1 × 10^7^ CFU/mL LcS. Example is from one experiment but representative of 3 independent experiments with similar results. (b) Summary graphs of all experiments, representing proportions of CLA^+^ T cells stimulated by 3% control and UC-DC conditioned with increasing doses of LcS (*n* = 3). One-way ANOVA was applied; *P* value <0.05 was considered statistically significant (*P < 0.05, **P < 0.01). (c) CLA/*β*7 dot plots of dividing T cells, following stimulation by 3% control DC, control DC + 1 × 10^7^ LcS, or UC-DC (no LcS). Example is from one experiment but representative of 3 independent experiments with similar results. Numbers over each dot plot represent proportion of dividing T cells expressing *β*7 only, *β*7 and CLA, CLA only, or either *β*7 or CLA. (d) Summary graph of all experiments (*n* = 3) representing the proportion of dividing T cells (stimulated by 3% DC in all cases) expressing CLA only (i.e., *β*7 negative), out of total CLA^+^ dividing T cells. Paired *t*-test was applied; *P* value <0.05 was considered statistically significant (*P < 0.05, **P < 0.01).

LcS conditioning of DC had differential effects in healthy controls compared with UC, on DC ability to imprint homing properties on stimulated T cells. In healthy controls, LcS conditioning enhanced DC ability to induce a skin-homing profile on T cells, significantly increasing the proportion of stimulated T cells expressing CLA, in a dose-dependent fashion (Figures 4(a) and 4(b)). However, in UC, CLA expression on T cells was already enhanced (Figures 2(a) and 2(b)), and LcS conditioning had no further effects on DC ability to enhance CLA expression on T cells (Figure 4(b)). LcS conditioning had no effect on DC ability to induce CCR4 expression in either healthy controls or UC patients (data not shown).

CLA expression on T cells was enhanced upon stimulation by both untreated UC-DC and LcS-conditioned (control) DC. Induction of CLA on T cells stimulated by LcS-conditioned DC from controls was in conjunction with gut-homing marker *β*7. However, CLA induction by untreated UC-DC was on the *β*7 negative fraction of T cells (Figure 4(c)). Thus, the proportion of CLA^+^ *β*7^−^ T cells within the total CLA^+^ dividing T cell pool was significantly greater upon stimulation with UC-DC, compared to LcS (1 × 10^7^ CFU/mL) conditioned (control) DC (Figure 4(d)).

#### 3.2.3. LcS Conditioned DC to Induce TGF*β* Production by T Cells in Healthy Controls but Not UC Patients

**Figure 5:**
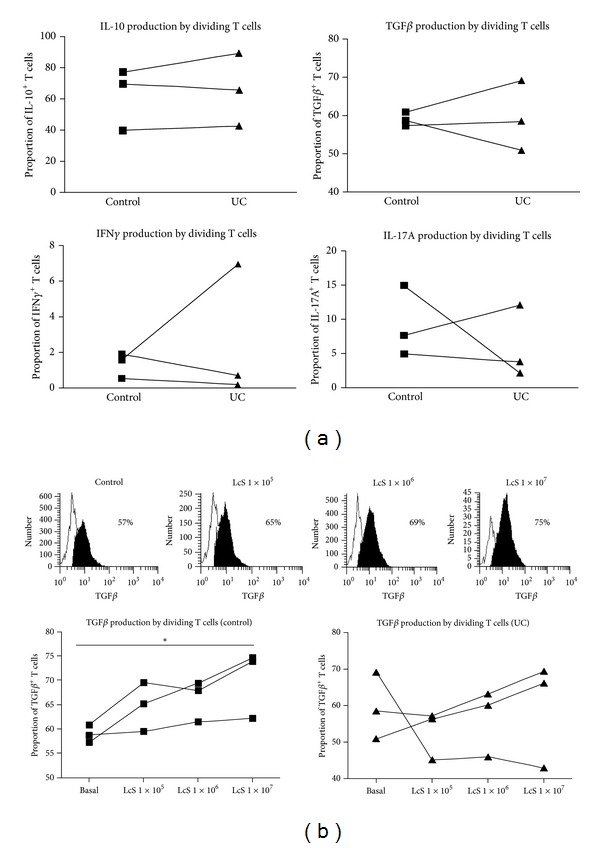
Intracellular cytokine production by stimulated T cells. (a) Summary graphs of all experiments representing proportions of T cells stimulated by 3% control and UC-DC producing TGF*β*, IL-10, IFN*γ*, and IL-17 (*n* = 3). Paired *t*-tests were applied; *P* value <0.05 was considered statistically significant. (b) Histograms of TGF*β* expression by dividing T cells, following stimulation by 3% control (no LcS) DC or control DC + 1 × 10^5^/1 × 10^6^ or 1 × 10^7^ CFU/mL LcS. Example is from one experiment but representative of 3 independent experiments with similar results. Summary graphs of all experiments (*n* = 3) representing the proportion of dividing T cells (stimulated by 3% DC in all cases) producing TGF*β*. DC were from healthy controls (left graph) or UC patients (right graph). One-way ANOVA was applied; *P* value <0.05 was considered statistically significant (*P < 0.05).

LcS conditioning of DC also had differential effects on DC ability to induce cytokine production by stimulated T cells, in controls compared with UC patients. Although there were differences within individual experiments between the ability of control DC and UC-DC (both untreated) to induce cytokine production by T cells (IL-10, TGF*β*, IFN*γ*, and IL-17A were measured), overall there were no significant differences (Figure 5(a)). However, TGF*β* production by T cells was significantly increased, in a dose-dependent manner, when DC were conditioned with LcS in healthy controls but not in ulcerative colitis (Figure 5(b)).

## 4. Discussion

We demonstrate for the first time that human circulating DC from UC patients exhibit a restricted stimulatory capacity for allogeneic T cells, and these DC induce a specific skin-homing profile on stimulated T cells that DC from healthy controls do not. Our data support studies demonstrating dysregulated DC function in IBD [11, 15, 16] and, furthermore, demonstrate systemic immune dysregulation in IBD patients rather than at mucosal sites only. The occurrence of extraintestinal manifestations (EIM) associated with IBD indicates that IBD is indeed a systemic disease, and our data provide an explanation for the occurrence of EIM affecting the skin [17]. Conditioning UC-DC with probiotic strain LcS restored their stimulatory capacity, reflecting that of control DC. LcS had differential effects on DC in healthy controls and UC on DC ability to imprint specific homing profiles on stimulated T cells, and to induce cytokine production by T cells. This is the first study, to our knowledge, to investigate the effects of probiotic bacteria on migratory properties of immune cells. Our data supports studies demonstrating multifunctional immunoregulatory activities of LcS, depending on the responding cell types and the local microenvironment [38].

LcS conditioned control DC, but not UC-DC, to imprint skin-homing molecule CLA on stimulated T cells. However, unlike the skin-homing profile induced on T cells by UC-DC, CLA expression induced via LcS conditioning was in conjunction with gut-homing molecule *β*7, suggesting induction of a multihoming profile. The differential effects of LcS on control and UC-DC were further demonstrated by the induction of TGF*β* production by T cells stimulated with LcS-conditioned DC in controls but not UC patients. These data suggest that effects of LcS exerted on human DC are flexible, depending on the responding cell types and the local cytokine environment. The restoration of UC-DC stimulatory capacity by LcS suggests that LcS may partly contribute to restoration/maintenance of homeostasis.

LcS may also confer homeostatic properties at intestinal sites (e.g., via oral administration) which could be beneficial in IBD; gut DC play a central role in immune homeostasis in the gut [39] and exhibit tolerogenic properties [15]. Alterations occur in gut DC in IBD [15, 16], leading to loss of tolerance in the gut and dysregulated immune responses to the colonic microbiota, a major contributing factor in the onset of IBD [11]. Restoration of homeostatic properties of gut DC by LcS at *intestinal *sites may account for the reported efficacy of LcS in UC [32]. However, the local microenvironment and responding cell types differ dramatically in the circulation and the gut, for example, gut DC are conditioned by intestinal epithelial cells and epithelial cell-derived products to adopt their tolerogenic function [40–42]. Future studies will determine *in vitro *effects of LcS on gut DC and also on epithelial cell conditioning of gut DC.

Although the definition of probiotics involves *live *microorganisms (which when administered in adequate amounts confer health benefit on the host) [2], our data demonstrates immunomodulation by *heat-killed *bacteria; furthermore, we demonstrated no significant differences between live and HK LcS regarding their ability to enhance activation marker expression on blood-enriched DC from healthy controls. These data support studies demonstrating immunomodulation by probiotic bacterial products, including the ability of probiotic bacterial DNA to induce regulatory IL-10 production by human peripheral blood mononuclear cells [43] and dendritic cells [44] and the ability of sonicated probiotic bacteria to induce marked anti-inflammatory effects on blood and intestinal DC. Furthermore, our recent studies have demonstrated that an immunomodulatory peptide secreted by *Lactobacillus plantarum* mediates some of the molecular dialogue between intestinal bacteria and DC, inducing immunoregulatory effects in both blood and intestinal DC *in vitro* [45].

IBD is associated with a variety of EIM, with up to a third of patients developing cutaneous manifestations including erythema nodosum (EN) and pyoderma gangrenosum (PG) [17]. The causes of EIM of IBD are poorly understood, but it has been suggested that compartmentalisation of inflammatory processes to different organs (e.g., intestine, skin, and liver) may be linked to homing and trafficking of immune cells. For example, CCL25, the ligand for gut-homing receptor CCR9, is expressed on epithelium in both the liver and the small intestine [18]. Dysregulation of lymphocyte trafficking plays a key role in IBD pathogenesis [19–22, 46] and IBD therapeutics have previously demonstrated efficacy by abrogating trafficking of effector cells to intestinal sites [47–51]. However, we demonstrate in this study that skin-homing markers CLA and CCR4 are aberrantly expressed on *β*7^−^ T cells stimulated by UC-DC, providing an explanation for the occurrence of EIM affecting the skin and supporting previous studies demonstrating that conditioning DC with supernatants from culture of colonic biopsies from UC patients enables them to imprint a skin-homing phenotype on stimulated T cells [52]. Blocking trafficking of effector cells to *cutaneous sites* in patients with EIM of IBD may also be of therapeutic benefit. 

Although there were no significant effects of LcS on DC ability to induce T cell cytokine production in UC overall, effects of LcS were variable between individual experiments, depending on whether production of particular cytokines was increased or decreased compared to T cells stimulated by control DC (data not shown). These data also suggest restoration of a “normal” phenotype and support a multifunctional immunoregulatory role for LcS, returning dysregulated immune functions to the original normal state when the host becomes either immunocompromised or excessively activated [38]. Indeed, LcS can have either pro- or anti-inflammatory effects in human intervention studies [32, 53, 54] and *in vitro *studies [55–57] depending on the context.

In summary, our data demonstrate systemic alterations in immune cells in UC, specifically a dysregulated DC function. Our data provides an explanation for the occurrence of EIM of the skin in UC patients and suggests that the probiotic strain LcS has multifunctional immunoregulatory activities on DC, depending on the disease state and the inflammatory environment. Our data supports studies demonstrating probiotic bacterial products, rather than live bacteria, are capable of inducing immunoregulatory effects. The reported therapeutic effects of LcS and other probiotic *Lactobacilli *strains in UC [32, 58] may be partly due to promotion of homeostasis, restoring the dysregulated functions of immune cells.
